# Prevalence of Parkinson’s disease across North America

**DOI:** 10.1038/s41531-018-0058-0

**Published:** 2018-07-10

**Authors:** C. Marras, J. C. Beck, J. H. Bower, E. Roberts, B Ritz, G. W. Ross, R. D. Abbott, R. Savica, S. K. Van Den Eeden, A. W. Willis, CM Tanner, on behalf of the Parkinson’s Foundation P4 Group

**Affiliations:** 1 The Morton and Gloria Shulman Movement Disorders Centre and the Edmond J Safra Program in Parkinson’s Research, Toronto Western Hospital, University of Toronto, Toronto, ON Canada; 2 The Parkinson’s Foundation, New York, NY USA; 3 New York University, New York, NY USA; 4 Department of Neurology, School of Medicine, Mayo Clinic, Rochester, MN USA; 5 Public Health Institute, Oakland, CA USA; 6 Department of Epidemiology, UCLA Fielding School of Public Health and UCLA Geffen School of Medicine, Los Angeles, CA USA; 7 Department of Environmental Health, UCLA Fielding School of Public Health and UCLA Geffen School of Medicine, Los Angeles, CA USA; 8 Department of Neurology, UCLA Fielding School of Public Health and UCLA Geffen School of Medicine, Los Angeles, CA USA; 9 Veterans Affairs Pacific Islands Health Care System, Honolulu, HI USA; 10 Center for Epidemiologic Research in Asia, Shiga University of Medical Science, Otsu, Japan; 11 Division of Research, Kaiser Permanente Northern California, Oakland, CA USA; 12 Department of Neurology, University of Pennsylvania Perelman School of Medicine, Philadelphia, PA USA; 13 Department of Biostatistics, Epidemiology and Informatics, University of Pennsylvania Perelman School of Medicine, Philadelphia, PA USA; 14 Department of Neurology, University of California – San Francisco & PD Research Education and Clinical Center, San Francisco Veterans Affairs Health Care System, San Francisco, CA USA

## Abstract

Estimates of the prevalence of Parkinson’s disease in North America have varied widely and many estimates are based on small numbers of cases and from small regional subpopulations. We sought to estimate the prevalence of Parkinson’s disease in North America by combining data from a multi-study sampling strategy in diverse geographic regions and/or data sources. Five separate cohort studies in California (2), Minnesota (1), Hawaii USA (1), and Ontario, Canada (1) estimated the prevalence of PD from health-care records (3), active ascertainment through facilities, large group, and neurology practices (1), and longitudinal follow-up of a population cohort (1). US Medicare program data provided complementary estimates for the corresponding regions. Using our age- and sex-specific meta-estimates from California, Minnesota, and Ontario and the US population structure from 2010, we estimate the overall prevalence of PD among those aged ≥45 years to be 572 per 100,000 (95% confidence interval 537–614) that there were 680,000 individuals in the US aged ≥45 years with PD in 2010 and that that number will rise to approximately 930,000 in 2020 and 1,238,000 in 2030 based on the US Census Bureau population projections. Regional variations in prevalence were also observed in both the project results and the Medicare-based calculations with which they were compared. The estimates generated by the Hawaiian study were lower across age categories. These estimates can guide health-care planning but should be considered minimum estimates. Some heterogeneity exists that remains to be understood.

## Disease prevalence: Measuring the rising tide

A large study that combines data from five different projects in four different regions across North America provides an updated estimate of the prevalence of Parkinson’s disease (PD). Connie Marras at Toronto Western Hospital in Canada and colleagues found that PD prevalence among individuals over 45 years of age is higher among men than women and that it increases with age in both sexes. They estimate that the overall prevalence of PD is 572 per 100,000 and that in the US in 2010 there were 680,000 individuals with PD. As life expectancy increases this number is projected to increase to over one million by 2030. These figures, which the authors note should be considered minimum prevalence estimates, warn of the impact that PD will have on North America’s healthcare systems in the near future.

## Introduction

Prevalence estimates of disease are important for public health planning. The upcoming demographic shifts toward older individuals in western nations have led to major efforts to project the health-care burden over the coming decades, particularly for diseases for which incidence rises considerably with age, such as Alzheimer’s disease and Parkinson’s disease (PD). The projected increases in dementia have been referred to as a “Rising tide,” emphasizing the sheer volume of this problem and warnings of the public health challenges of caring for these individuals have been issued.^1^ PD presents similar challenges as its prevalence in the world’s most populous nations has been projected to more than double between 2005 and 2030.^2^ These estimates do not account for changes in exposures that may further contribute to increased incidence or prevalence.^3^

A recent systematic review and meta-analysis examined the prevalence of PD worldwide as estimated by studies performed since 1985. Included in the review were studies that used door-to-door ascertainment or random population sampling followed by physical examination.^4^ Point estimates of age-specific prevalence across regions varied widely, and confidence intervals were broad, limiting the interpretability of these data for public health planning. Furthermore, only two such studies were identified in North America since 1985, both in Canada identifying 2 and 4 PD cases each.^5,6^ An earlier study of PD prevalence in Copiah County, Mississippi, USA conducted in 1978 is commonly used as the estimate of prevalence for PD in the US, although its calculations were based on only 26 cases of idiopathic PD ascertained from a small area.^7^ More recent estimates based on more robust data from a wider sampling frame are needed.

Commonly, medical records or health system claims data are used to estimate PD prevalence. The limitations of this approach with respect to diagnostic misclassification notwithstanding, an advantage of such studies is their size, often using data on millions of individuals to deliver more precise estimates than can be produced by studies that contact people directly. North American estimates of PD prevalence from such studies have varied widely and have noted geographical variation.^8–13^

Beginning in 2014, the Parkinson’s Foundation established the Parkinson’s Foundation Parkinson’s Prevalence Project (P4) to coordinate epidemiological investigations of prevalence conducted at disparate sites throughout North America. The P4 Project combines data from five different recent or current projects covering four different regions across North America and compares these to prevalence estimates generated using nationwide data from the US to provide an updated and more precise estimate of PD burden. Specifically, we aimed to address the following questions:

1. Is the prevalence of PD uniform throughout North America or does it vary by study and/or geography?
2. To the extent that these data can be considered consistent, what meta-estimates of prevalence do they provide?

## Results

**Fig. 1:**
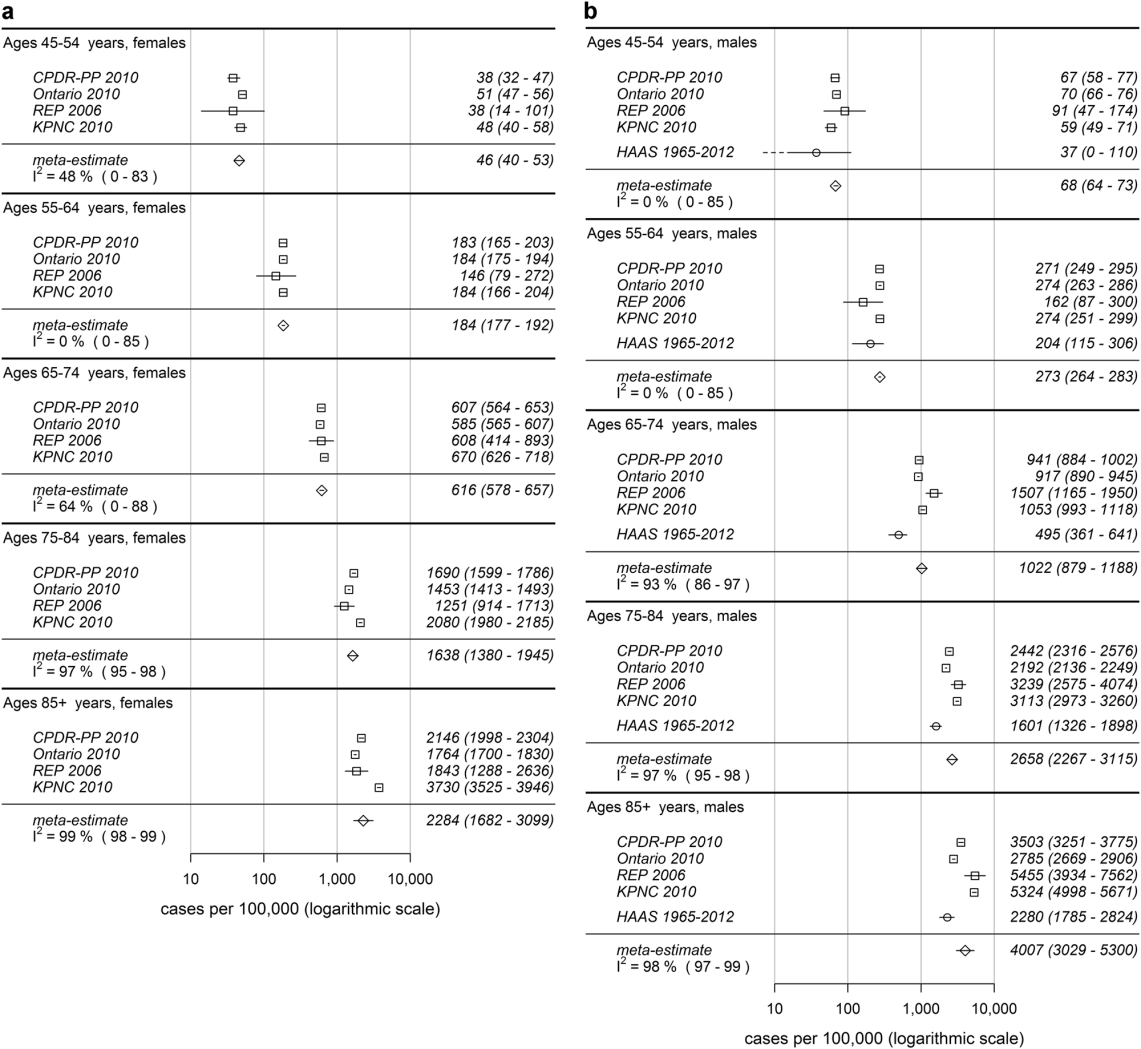
Prevalence of PD aged ≥45 years by age group and sex: **a** Females. **b** Males. HAAS Honolulu-Asia Aging Study, REP Rochester Epidemiology Project, CPDR-PP California Parkinson’s disease Registry-Pilot Project, KPNC Kaiser Permanente Northern California Integrated Health Care System. In each row, the squares or diamonds are centered on the point estimate of the prevalence and whiskers represent 95% confidence intervals. Point estimates for HAAS are indicated by circles instead of squares because the meta-estimates exclude the HAAS study due to methodologic differences between this and the other studies

The populations studied (aged ≥45 years) ranged in size from 8006 (Honolulu-Asia Aging Study (HAAS)) to 5,525,787 (Ontario). The number of cases identified by project ranged from 207 to 28,065. Table 1 shows the case numbers, denominators, and prevalence estimates for each project by sex. The supplementary table provides prevalence further broken down by age group. Figure 1 displays the prevalence estimates closest to 2010 for each project and provides the meta-analytic estimate for each age group by sex. The prevalence within age strata below the age of 65 years for males and below the age of 75 years for females have confidence intervals for *I*^2^ containing nearly its entire possible range, which implies that our results do not contain enough information to rule heterogeneity in or out. For older ages, heterogeneity is demonstrated. The estimates were higher in men than in women and rose with age in both sexes. Using Kaiser Permanente Northern California Integrated Health Care System (KPNC), Ontario, Rochester Epidemiology Project (REP), and California Parkinson’s disease Registry-Pilot Project (CPDR-PP) data, the combined prevalence for men and women aged >45 years standardized by age and sex to the US population according to the 2010 US census was 572 per 100,000 (95% confidence intervals 537–614). For females, the prevalence was 488 (444–543), and for males 667 (612–732). Based on published data, we calculated the prevalence of parkinsonism as estimated by the Copiah County study^7^ standardized to the US population (2010) to be 301 per 100,000 (Table 2). Using our age- and sex-specific meta-estimates and applying these to the US population according to the 2010 census, we would expect 680,000 cases of PD in the US among individuals aged ≥45 years. Given the projected population structure for future years and assuming stable age and sex-specific prevalences, we estimate that this number would rise to 930,000 cases in 2020 and 1,238,000 by 2030.

**Table 1:** Prevalence of Parkinson’s disease by study, year, and sex

Study	Year	Population	Number of cases	Age-standardized rate ^a^ (95% CI)
*Females, 45+ years*				
CPDR-PP	2010	693,990	3204	462 (456–468)
KPNC	2010	654,545	4081	623 (617–631)
Ontario	2010	2,892,521	12,972	448 (446–451)
REP	2006	26,394	109	413 (385–442)
*Males, 45+ years*				
CPDR-PP	2010	644,807	3728	578 (571–585)
KPNC	2010	560,338	4484	800 (791–809)
Ontario	2010	2,633,266	15,093	573 (570–577)
REP	2006	22,881	186	813 (769–859)
HAAS	1965–2012	8006	207	380 (308–464)

*REP* Rochester Epidemiology Project, *CPDR-PP* California Parkinson’s disease Registry-Pilot Project, *KPNC* Kaiser Permanente Northern California, *HAAS* Honolulu-Asia Aging Study

^a^Standardized to US 2010 population based on 5-year age groups

**Table 2:** Prevalence of PD in Copiah County, Mississippi in 1978 standardized to US population 2010^a^

Sex	Age, years	Denominator	Cases	US population 2010	Expected cases	Age-standardized prevalence/100,000
Male	40–64	2473	4	50,137,484	81,096	
	65–74	948	4	10,096,519	42,601	
	75+	472	5	7,266,441	76,975	
Female	40–64	3016	3	52,242,925	51,966	
	65–74	1235	8	11,616,910	75,251	
	75+	781	7	11,288,114	101,174	
Total			31	142,648,393	429,063	301

^a^Based on previously published work by others.^7^ Includes possible and definite cases with 5 of the 31 cases designated as post-encephalitic parkinsonism. These were not removed because age-specific numbers were not available by diagnosis

**Fig. 2:**
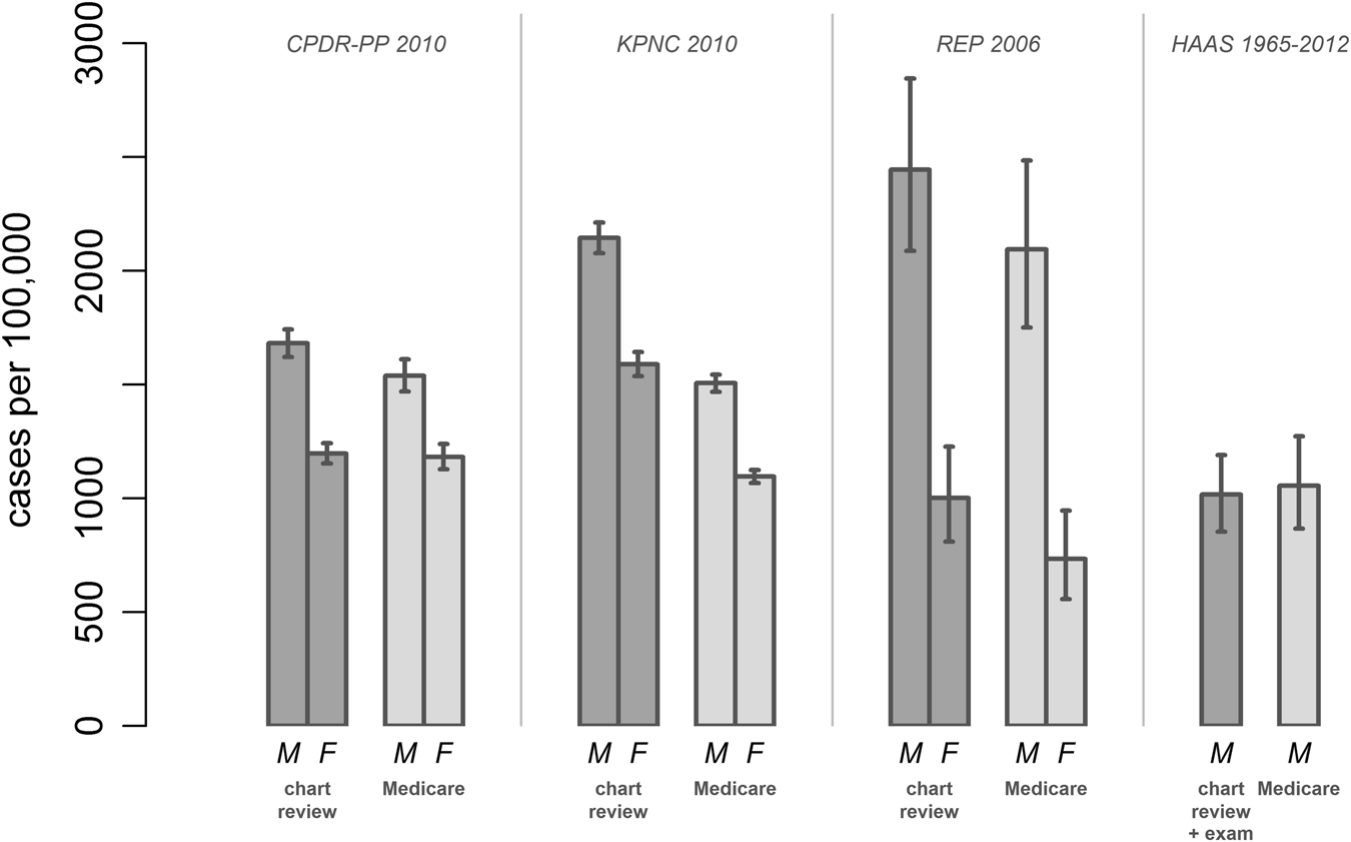
Prevalence estimates by study compared to Medicare data from the corresponding counties. The corresponding counties were as follows: For California PD Registry Pilot Project: Fresno, Kern, Santa Clara, and Tulare, for Rochester Epidemiology Project: Olmsted, for KPNC: Alameda, Amador, Contra Costa, El Dorado, Fresno, Kings, Madera, Marin, Mariposa, Merced, Napa, Placer, Sacramento, San Francisco, San Joaquin, San Mateo, Santa Clara, Santa Cruz, Solano, Sonoma, Stanislaus, Sutter, Tulare, Yolo, and Yuba counties, for HAAS: Honolulu

Figure 2 shows the prevalence estimates for the studies of California, Olmsted County, Minnesota, and Hawaii juxtaposed with the prevalence estimates derived from US Medicare data from the corresponding counties. Because Medicare beneficiaries are aged ≥65 years, we employ the portions of study subjects matching this age restriction. For two studies (CPDR-PP and HAAS), prevalence rates were statistically indistinguishable from the Medicare estimates. In contrast, the Rochester Epidemiology Project (Olmsted County) identified 14–27% more cases, and the KPNC approximately 30% more. However, the pattern of variation between regions is reproduced regardless of whether Medicare data or the P4 study data are used (Fig. 2).

## Discussion

Our multi-study, multi-regional approach provides current estimates for PD prevalence among individuals over the age of 45 years, which overall is 572/100,000. The estimates from the individual studies are generally similar, and the meta-estimate confidence intervals are narrow, reflecting the large size of the component studies. Despite the overall similarity, there is evidence for heterogeneity, particularly a lower estimated prevalence from the HAAS study conducted on Japanese American men in Hawaii. HAAS has, since 1991, complemented its medical record-based case-finding with an in-person screening examination of participating individuals. This method would be expected to minimize missing cases, and as none of the other studies undertook unselected screening and examination of participants, one would expect the ascertainment in the HAAS study to be the most complete. In addition, loss to follow-up rates are very low.^14^ These observations suggest that the lower prevalence estimate reflects differences in environmental or genetic risk factors or could relate to the timing of the study that extends from 1965 to 2012. Incidence rates in the HAAS cohort reported in prior studies were reported as being comparable to rates in European and the US populations and were higher for Asians living in Asia.^14^ Interestingly, in a recent systematic review of PD prevalence studies using random sampling methods or door-to-door ascertainment with case verification through in-person examination, individuals 70–79 years of age in Asia were found to have a significantly lower prevalence of PD (646 per 100,000) compared with individuals of the same age in Europe, North America, and Australia (1602 per 100,000; *P* < 0.05).^4^ Using Medicare data, a lower prevalence in Asian individuals living in the United States than White or Hispanic individuals has been found^8^ and a non-significantly lower incidence in individuals of Asian ancestry has also been found using KPNC data from northern California.^15^ These findings are consistent with our own.

Despite the fact that the P4 prevalence estimates other than HAAS are similar to each other, our sample sizes are large enough to document significant heterogeneity and this heterogeneity is mirrored in the subgroup over age 65 years by corresponding variations in estimates based solely on Medicare records when adjusting for age and sex. We find this suggestive that—even within North America—the prevalence of PD that has come to medical attention does vary by region. Geographic variations in PD prevalence within the US have also been reported previously using solely Medicare data: Willis et al.^1^ found two- to ten-fold variations in PD prevalence between US counties. Whether or not these differences reflect variations in health-care seeking practices or access to care (including access to neurologists) or regional differences in environmental or genetic determinants of disease cannot be determined by our study and remain an important question for future research.

The heterogeneity must be kept in mind when expressing the prevalence of PD in North America as a single number. The meta-estimate is generated from projects sampling from a few disparate regions and we did not account for the heterogeneity that likely exists in other regions of North America. There is additional evidence for ethnic, socioeconomic, and geographic variation in PD prevalence in North America. A study of continental American and Alaskan natives^10^ reported a prevalence of 822/100,000 over age 40 years standardized to the 2000 US population, which is higher than most estimates from other places and ethnic groups worldwide. As previously mentioned, analysis of the Medicare nationwide database found a higher prevalence in Whites and Hispanics than in Black or Asian individuals.^8^ That same study found Copiah County, Mississippi, USA to have one of the lowest prevalence rates in the country and also revealed other geographic variations within the US. The geographic areas from which our data are drawn have a lower proportion of individuals of Black race than the US overall. Thus not taking these racial distributions into account would result in a higher estimate of PD prevalence for the entire US. Despite this potential bias, we believe that our estimates underestimate the total burden for reasons explained further below.

Other variations in PD prevalence have also been observed. Using health-care claims, PD prevalence in the Canadian province of Manitoba in both rural and urban areas was higher in areas with lower average income.^11^ Rates of hospital discharges for PD across Canada have also been reported to vary significantly, suggesting that PD prevalence was higher in Western provinces.^12^ The reasons for these observed variations need to be understood. They support the need for a sampling scheme with wide geographic and socioeconomic variation if a representative sample is going to be obtained. However, our prevalence estimates are consistent enough to support the use of our meta-estimates excluding the HAAS for use in public health discussion and planning at least until more exhaustive region-specific studies within North America become available.

Previous PD prevalence estimates in North America have varied widely. The now historical study of PD prevalence in Copiah County^7^ ascertained cases of PD through door-to-door contact, conducting screening interviews by a non-medical interviewer followed by neurological evaluation by a physician and an extensive multi-source data-gathering method in those who screened positive.^16^ Despite this careful case ascertainment, the age- and sex-standardized prevalence estimate in individuals over age 45 years, standardized to the US 2010 population is 301/100,000, smaller than our estimate of 572. The authors of the Copiah County study appropriately cautioned readers on assuming generalizability of their findings beyond the geographical boundaries of Copiah County and emphasized the usefulness of their work as providing inter-racial comparisons.

Several other regional studies of PD prevalence within North America have been published. Using the health-care administrative data sources in Ontario, Canada, the average prevalence of PD in 1992–1998 was estimated to be 363/100,000 in men and 324/100,000 in women.^13^ Direct comparison is difficult, however, because this estimate was derived using different case identification criteria, included the entire lifespan (whereas our estimates are restricted to individuals over age 45 years), and these are estimates that were adjusted to the Ontario population structure. A prevalence of 144/100,000 in the Canadian province of British Columbia was reported using drug-tracer methodology, which would only detect treated PD.^9^ The Canadian Community Health Survey found that 200/100,000 of Canadians living in private households self-reported a diagnosis of PD.^17^ Although generally lower than our estimates, variations in ascertainment methods could account for these differences.

The validity of our estimates relies on accurate and complete case ascertainment. Our estimates likely underestimate the true prevalence for several reasons: (1) Except for cases in HAAS, PD cases in the other studies included were all identified through accessing medical records or claims data of health-care systems and thus would miss those not seeking care, seeking care from other types of health-care providers, or without access to care. (2) Ascertainment through assigned diagnostic codes is inevitably incomplete due to incomplete or inaccurate coding. For example, the sensitivity of the ascertainment algorithm for cases in Ontario, the largest of the P4 projects, is estimated to be approximately 72%. (3) Some studies could not conduct complete ascertainment: for example, in the CPDR-PP not all providers were contacted due to limited resources and CPDR-PP and Medicare estimates do not include cases treated by providers whose data were not shared owing to strict privacy or administrative rules. Thus our estimates are best considered minimum prevalence estimates. At the same time, we must acknowledge that without individual case validation, which was not possible in several of our datasets, there is risk of both false-positive and false-negative determinations of case status. Despite many sources of variation, however, we derived fairly consistent estimates of prevalence among the component projects.

The projections of PD case numbers over the next 12 years assume stable prevalence within age and sex strata. However, if incidence rises or mortality from PD lessens, then prevalence would rise and our projections would underestimate the case burden above and beyond the issues of incomplete ascertainment mentioned above. There is evidence that incidence may be increasing^18^ and this is consistent with the fact that our lowest prevalence estimates came from the HAAS project, which spanned earlier years than the other projects in P4.

Our projected minimum estimates of PD case burden, rising to more than one million people in the US by 2030, highlight the growing importance of optimizing care and treatment for people with PD, lessening the burden of care on the caregivers, and easing the strain on health and elder care systems. Furthermore, our data and data from other studies suggest regional variation that deserves to be studied in order to reveal whether or not this variation stems from differences in susceptibility to the disease or differences in access to or utilization of health-care services. Accurate estimates of PD incidence and mortality in different geographic regions are also needed to assist in answering these questions.

## Methods

### Data collection

Prevalence was estimated for individuals aged ≥45 years from 5 different projects undertaken in 4 different regions of USA and Canada: (1) Ontario, Canada, (2) Olmsted County, Minnesota, USA: The REP,^19^ (3) California, USA: KPNC^15^ and the CPDR-PP, and (4) Honolulu County, Hawaii, USA: The HAAS.^14^ In addition, data from the US Medicare program, which provides health insurance to 98% of the population aged ≥65 years was used as a complementary data source. The study populations and case ascertainment methods are described in Table 3. For comparison purposes, data from Copiah County, Mississippi, USA were taken from the published literature.^7^

**Table 3:** Study populations and case ascertainment methods

Study	Honolulu-Asia Aging Study	Ontario, Canada	Kaiser Permanente Northern California	Rochester Epidemiology Project	California PD registry project	US Medicare
Base population	8006 Japanese-American men born 1900–1919, living in Honolulu county, Hawaii, USA at baseline in 1965 and participating in the longitudinal Honolulu Heart Program	Residents of Ontario, Canada; all are provided health care paid for by the provincial government	Members of the Kaiser Permanente Northern California, a closed integrated health-care delivery system providing health insurance and health care to 25–30% of the population of Northern California^a^	Residents of Olmsted county, Minnesota, USA	Residents of Kern, Tulare, Fresno, Santa Clara counties, California, USA	Residents of USA aged ≥65 years who use Medicare as their health-care insurer and whose insurance claims are released to Medicare^b^
Ascertainment method(s)/data source	Pre-1991: Hospitalization records, outpatient medical records, Post-1991: Screening in-person exam by trained research technician, positive cases examined by neurologist	Ontario Health-care administrative databases recording all inpatient and outpatient physician encounters	Medical record ascertainment that combined inpatient and outpatient diagnostic, pharmacy, treatment, and physician type^15^	Electronic screening for 53 H-ICDA codes for PD, parkinsonism, tremor, PSP, MSA, other extrapyramidal syndromes, non-specific neuro-degenerative diseases, followed by manual medical record review by neurologist^28^	Neurologists and large group practices asked to report all patients with ICD-9 code of PD (332) or other parkinsonism (332.1, 333.0, or 331.82). Trained abstractors manually extracted relevant elements of medical record	Medicare administrative claims database
Diagnostic criteria	Consensus diagnosis by movement disorders experts using hospitalization, outpatient neurologist records, and additionally after 1991 study screening examination and study neurologist’s standardized examination and Ward and Gibb criteria^29^	One hospitalization record or two outpatient visits with an assigned ICD diagnosis of PD (332 or G20) in the administrative record^30^	Algorithm that combines number of PD diagnoses, expertise of the physician making the diagnoses, and treatment	The presence of two of four cardinal signs: resting tremor, bradykinesia, rigidity, and impaired postural reflexes, without a known secondary cause, documented levodopa unresponsiveness or other atypical features^28^	ICD-9 code for PD (332). If more than one parkinsonism code was reported, manual medical record review by a movement disorder neurologist (CMT) to assign the most likely diagnosis	One ICD code for PD (332.0) and no atypical or secondary parkinsonism codes
Case definition validation method(s), if any	None	Medical record review. Sensitivity 72%, specificity 99%^30^	None	Clinicopathologic concordance 87% in 60 individuals^31^	A minimum of 10% validation using standardized chart abstraction protocol	None

*H-ICDA* Hospital adaptation of ICD. 53 H-ICDA diagnostic codes: 7 codes for PD, 12 for parkinsonism, 10 for tremor, 8 for other extrapyramidal symptoms, 6 for nonspecific neurodegenerative diseases, 5 for multiple system atrophy, and 5 for progressive supranuclear palsy

^a^Members are representative of the population of Northern California with respect to age, sex, and race/ethnicity and slightly less likely to have very low or very high income^27^

^b^While Medicare provides health insurance to 98% of the population aged ≥65 years, some individuals choose third-party medical insurance coverage and some health-care organizations or reimbursement programs do not release their claims data to Medicare due to privacy regulations or for other reasons

To be designated as a prevalent case in all projects except HAAS, the individual must have been alive and residing in the geographic area on the prevalence day or during the prevalence year. Diagnostic criteria (Table 3) needed to be met prior to the prevalence day or during the prevalence year in all projects except the REP where symptom onset before the prevalence day was sufficient to satisfy the designation of prevalent. The ascertainment years were 2010 for Ontario, KPNC, and CPDR-PP and 2006 for REP. For the cohort study, HAAS, a prevalent case was defined as having PD within the period (i.e., age stratum) of interest at any time between 1965 and 2012.

The work was approved by the following research ethics committees:

Ontario, Canada: The Research Board at Sunnybrook Health Sciences Centre, Toronto.

CDPR-PP: The ascertainment activity was reviewed by the State of California IRB and determined to be public health surveillance. Use for research was approved as part of the PRIDE project, approved by the State of California IRB and the University of California at San Francisco IRB. The University of California at Los Angeles IRB also approved the CPDR-PP.

KPNC: The University of California at San Francisco IRB.

HAAS: Kuakini Medical Center, Honolulu, HI, and Veterans Affairs Pacific Islands Health Care System, Honolulu, HI Institutional Review Boards.

REP: The Institutional Review Boards of Mayo Clinic and Olmsted Medical Center, Rochester, MN.

### Statistical analysis

Using prevalence data from the individual studies, we employed the metafor^20^ package within the R programming environment^21^ to fit random-effects models for each sex and 10-year age stratum 45–54 through 85+. We represent heterogeneity using the *I*^2^ statistic of Higgins and colleagues;^22^ this statistic describes the percentage of variation across studies that appears to be due to study heterogeneity rather than chance. 95% confidence intervals for the *I*^2^ statistic were calculated using published methods.^23^

For females and males, we apply the random-effects regression separately to each of our five 10-year age groups $i$, resulting in summary coefficients $\hat \beta _i$ such that the age-specific prevalence is $e^{\hat \beta _i}$. Similarly, our age-standardized meta-estimate is $\hat r = \mathop {\sum }\limits_i w_i\left( {e^{\hat \beta _i}} \right)$, where $w_i$ is the proportional representation of each age group in the 2010 US population^24^ such that $\mathop {\sum }\limits_i w_i = 1$. Confidence intervals for $\hat r$ were made by replacing $\hat \beta _i$ in the above formula with $\beta _i\sim N\left( {\hat \beta _i,\hat \sigma _i^2} \right)$, where $\hat \sigma _i^2$ is the variance of each coefficient $\hat \beta _i$. We report the 2.5th and 97.5th percentiles of $\hat r$ based on an arbitrarily large number of samples (1 million of each $\beta _i$). Estimates of the number of expected cases in 2020 and 2030 are calculated using the population projections for the entire nation of the US published by the US Census Bureau.^24^

Unique among the P4 efforts, HAAS is a longitudinal study following a fixed cohort of men over an extended period of time (1965–2012). The total number of years each subject was alive constitutes the number of person-years at risk (denominator), while the number of years over which a subject had a diagnosis of PD defines the case number within a period of interest (numerator). While common assumptions regarding the Poisson distribution of rare diseases can be applied to the other efforts to generate measures of uncertainty, HAAS data require a bootstrap resampling approach.^25^ As such, we report prevalence estimates from the HAAS cohort for comparison alongside those from other research efforts and do not incorporate them into the random-effects models to derive the summary estimates.

To compare prevalence estimates generated by the four P4 projects to those produced by US Medicare records, we calculated prevalence from Medicare data for each group of US counties included in the US P4 project catchment areas (see legend to Fig. 2 for the corresponding counties).

All rates are presented for males and females aged ≥65 years and standardized to the US 2010 population using 5-year age strata;^24^ confidence intervals are calculated using the method of Tiwari and colleagues.^26^

### Data availability

Data for this project are not owned by or under the control of the authors. Owing to either privacy regulations or data-sharing agreements, data cannot be made available.

### Code availability

Analytic code can be made available upon request to the corresponding author.

## Electronic supplementary material

Supplementary Information
