# Supplementary material for: Prevalence of Parkinson’s disease across North America
Source: NPJ Parkinsons Dis. 2018 Jul 10;4:21. doi: 10.1038/s41531-018-0058-0 (PMC6039505; doi:10.1038/s41531-018-0058-0)
Supplement: Supplementary file 1 — Supplementary Information [file 41531_2018_58_MOESM1_ESM.pdf]

eTable 1: Prevalence of PD by study, age group and sex

Group	Study, year	Denominator*	Cases*	Prevalence /100,000	95% CI /100,000
Women, 45-54					
	CPDR-PP 2010	265792	102	38	32-47
	KPNC 2010	218687	105	48	40-58
	Ontario 2010	1050338	51	51	47-56
	REP 2006	10543	4	38	14-101
Men, 45-54					
	CPDR-PP 2010	273684	182	67	58-77
	KPNC 2010	200300	118	59	49-71
	Ontario 2010	1048484	739	70	66-76
	REP 2006	9928	9	91	47-174
	HAAS 1965-2012	21,410	8	37	0-110
Women 55-64					
	CPDR-PP 2010	199924	366	183	165-203
	KPNC 2010	205864	379	184	166-204
	Ontario 2010	817224	1507	184	175-194
	REP 2006	6831	10	146	79-272
Men 55-64					
	CPDR-PP 2010	191816	520	271	249-295

	KPNC 2010	179737	492	274	251-299
	Ontario 2010	779878	2139	274	263-286
	REP 2006	6191	10	162	87-300
	HAAS 1965-2012	60750	124	204	115-306
Women 65-74					
	CPDR-PP 2010	118445	719	607	564-653
	KPNC 2010	122013	818	670	626-718
	Ontario 2010	513982	3008	585	565-607
	REP 2006	4275	26	608	414-893
Men 65-74					
	CPDR-PP 2010	104023	979	941	884-1002
	KPNC 2010	103846	1094	1053	993-1118
	Ontario 2010	461011	4227	917	890-945
	REP 2006	3848	58	1507	1165-1950
	HAAS 1965-2012	68101	337	495	361-641
Women 75-84					
	CPDR-PP 2010	74553	1260	1690	1599-1786
	KPNC 2010	75674	1574	2080	1980-2185
	Ontario 2010	351193	5102	1453	1413-1493
	Rochester 2006	3117	39	1251	914-1713

Men 75-84					
	CPDR-PP 2010	55646	1359	2442	2316-2576
	KPNC 2010	58368	1817	3113	2973-3260
	Ontario 2010	267872	5871	2192	2136-2249
	REP 2006	2254	73	3239	2575-4074
	HAAS 1965-2012	49724	796	1601	1326-1898
Women 85+					
	CPDR-PP 2010	35276	757	2146	1998-2304
	KPNC 2010	32308	1205	3730	3525-3946
	Ontario 2010	159784	2818	1764	1700-1830
	REP 2006	1628	30	1843	1299-2636
Men 85+					
	CPDR-PP 2010	19638	688	3503	3251-3775
	KPNC 2010	18088	963	5324	49985671
	Ontario 2010	76021	2117	2765	2669-2906
	REP 2006	660	36	5455	3934-7562
	HAAS 1965-2012	22630	516	2280	1785-2824

REP=Rochester Epidemiology Project, CPDR-PP= California Parkinson's disease Registry-Pilot Project, KPNC=Kaiser Premanente Northern California

*All denominator and case units are individuals except for the HAAS study, for which the units are person-years

eTable2: Statistical tests of heterogeneity for random effects models.

Sex	Age (years)	I^2 (95%-confidence interval)	Q	p-value
**Male**				
	45-54	0 (0 - 85)	4.2	0.2456
	55-64	0 (0 - 85)	2.8	0.4189
	56-74	93 (86 - 97)	29.4	0.0000
	75-84	97 (95 - 98)	178.3	0.0000
	85+	98 (97 - 99)	286.1	0.0000
**Female**				
	45-54	48 (0 - 83)	7.3	0.0621
	55-64	0 (0 - 85)	0.5	0.9101
	56-74	64 (0 - 88)	11.9	0.0077
	75-84	97 (95 - 98)	162.0	0.0000
	85+	99 (98 - 99)	474.9	0.0000
